# An organotypic model for investigating drug-radiation responses in the lung

**DOI:** 10.14440/jbm.2025.0080

**Published:** 2024-11-28

**Authors:** Maryam Alkadhimi, Anuradha Helen Manne, Yanyan Jiang, Marcus Green, Anderson Joseph Ryan

**Affiliations:** 1 Department of Oncology, University of Oxford, Oxford, OX3 7DQ, United Kingdom; 2 FastBiopharma, Watlington, OX49 5SW, United Kingdom

**Keywords:** DNA double-strand breaks, Lung, DNA-dependent protein kinase, Organotypic, Ionizing radiation, DNA repair

## Abstract

**Background:**

Established *in vivo* radiobiological models are commonly used to assess anti-tumor effects and normal tissue toxicity. However, these models have notable limitations, and additional models are necessary to gain a deeper insights into drug-radiation interactions.

**Objective:**

This study aimed to develop an organotypic *ex vivo* model by using precision-cut lung slices (PCLSs) to evaluate radiation-induced residual deoxyribonucleic acid (DNA) damage, both alone and in combination with a pharmacological inhibitor of DNA double-strand break (DSB) repair.

**Methods:**

Left lungs from female C57BL/6 mice were dissected, perfused with 4% low-gelling-temperature agarose, and sliced into 250 μm sections. Lung slices were then incubated *ex vivo* for up to 7 days. The slices were irradiated using ^137^Cs, either with or without a DNA-dependent protein kinase (DNA-PK) inhibitor (NU7441). Tissue sections were subsequently fixed and stained for γH2AX and 53BP1, which serve as histological markers of DNA DSBs.

**Results:**

The established conditions preserved tissue viability for up to 7 days and maintained structural integrity for 2 days. DNA damage, detected through γH2AX and 53BP1 staining, was consistent between lungs irradiated *ex vivo* and their counterparts irradiated *in vivo*. In the organotypic model, radiation alone in DNA-PK-deficient SCID mice and radiation combined with DNA-PK inhibition in C57BL/6 mice led to increased residual γH2AX and 53BP1 staining.

**Conclusion:**

This study demonstrates that residual DNA damage levels following ionizing radiation in lung tissue are comparable between *in vivo* and *ex vivo* tissue slices, suggesting that PCLSs serve as a valuable organotypic model for investigating the effects of drug-radiation combinations.

## 1. Introduction

Pre-clinical studies are commonly conducted to explore the effects of radiation on normal lung cells and tissues.^1^-^3^ While *in vitro* studies are essential for elucidating underlying mechanisms, they have notable limitations in evaluating normal tissue responses. In contrast, mouse lungs share similar tissue characteristics and physiology with human lungs, with early and late radiation-induced toxicities following timelines that largely parallel those observed in humans.^4^,^5^ However, mouse models also present challenges, such as difficulties in delivering a uniform dose exclusively to the lungs or target regions, primarily due to organ motion from rapid breathing and heartbeat.^6^ Therefore, while *in vivo* studies of drug-radiation effects on normal tissues closely mimic clinical conditions, the lack of precise control over experimental variables, including radiation dose distribution and tissue drug concentrations, highlights the potential utility of alternative models, which may also reduce the number of animals required for experiments.^7^

Organotypic models aim to accurately replicate the structure and function of tissues or organs in a natural, three-dimensional environment. Derived from living tissue, these models preserve many of the cellular interactions and physiological traits of the original organ, offering a more lifelike setting for studying biological processes than traditional two-dimensional cell cultures.^8^,^9^

In this study, we developed *ex vivo* precision-cut lung slices (PCLSs) as an organotypic model to investigate radiation and drug-radiation interactions.

## 2. Materials and methods

### 2.1. Ethical approval

All experiments were performed under the United Kingdom (UK) Home Office Project License PPL30/3395 following independent ethical review and approval.

### 2.2. *In vivo* irradiation

Mice were sedated by intraperitoneal injection of 100 µL solution containing 8 mg/mL Ketaset (Orion Pharma, UK) and 100 mg/mL Dormitor (Orion Pharma, UK) in saline. Once anesthetized, the mice were positioned in a Gulmay Medical RS320 X-ray irradiator (UK) set at 300 kV and 10 mA. Lead shields were used to cover the mice, leaving only a 1×2 cm area over the thorax exposed. The exposed thoracic region was irradiated with 10 Gy at a dose rate of 1.82 Gy/min with a total exposure time of 5 min and 30 s. Following radiation, each mouse received an intraperitoneal injection of 100 μL Antisedan (0.25 mg/mL stock concentration in saline; Orion Pharma, UK) and was placed in a 37°C recovery chamber for approximately 30 min to ensure full recovery from anesthesia.

### 2.3. *Ex vivo* irradiation

Tissue slices in six-well culture plates were exposed to ionizing radiation (γ rays) using a ^137^Cs irradiator (GSR D1, Gamma Service Medical GmbH, Germany) at a dose rate of 1.35 Gy/min.

### 2.4. Dosimetry

Dosimetry was carried out by Mark Hill and James Thompson from the Radiation Biophysics Group at the Department of Oncology, University of Oxford. Gafchromic EBT2 dosimetry film (ISP Technologies, USA) was exposed to radiation and subsequently scanned. The resulting optical density measurements were then converted to radiation doses using a calibration curve based on pre-determined irradiation doses.

### 2.5. Lung dissection

Female C57BL/6 or SCID mice (6–8 weeks old, sourced from Charles River, UK) were euthanized with a lethal dose of phenobarbitone, followed by cardiac removal. A midline incision was made from the abdomen to the chest to expose the trachea, which was carefully separated from surrounding connective tissues. A catheter was inserted into the trachea and secured with a suture. The lungs were inflated by perfusion with 1 mL of 4% low-gelling-temperature agarose (161-3111, Bio-Rad, UK) in phosphate-buffered saline (PBS) (37°C) through the catheter. Subsequently, the lungs were immediately dissected from the mouse, placed in PBS, and transferred to ice to allow the agarose to solidify.

### 2.6. PCLSs

The left lungs, inflated with solidified agarose, were immersed in a warm (37°C) 4% low-gelling-temperature agarose solution in PBS and then chilled in a refrigerator at 4°C for 15 min to solidify. Excess agarose surrounding the lung was trimmed away, and the lung-agarose sample was affixed to an epoxy-coated metal buffer tray using cyanoacrylate super glue. The sample was set on crushed ice for 10 min. For tissue slicing, a vibratome (VT1200S, Leica Biosystems, UK) was configured with the following settings: The blade was positioned at a 90° angle on the blade holder, with a 17° blade angle, amplitude of 3.00 mm, speed of 0.60 mm/s, and slice thickness of 250 μm, with slicing distance adjusted to lung size. The tray was filled with ice-cold PBS containing antibiotics (penicillin-streptomycin, 15070-063, Life Technologies, UK), and the lung was sliced accordingly. Individual 250 μm lung slices were placed on filters in separate wells of a 6-well plate filled with PBS and antibiotics. A control lung section was immediately fixed in 4% paraformaldehyde (28794.295, BDH Chemicals, UK) overnight after vibratome slicing, without cell culture, then transferred into 70% ethanol and stored at 4°C for up to 7 days before embedding in paraffin wax.

### 2.7. Preparation of tissue culture plates

Six-well tissue culture plates were prepared by placing Millicell filters (0.4 μm pore size, 30 mm height; Millipore, UK) into individual wells using sterilized tweezers. Each filter was moistened with 1 mL of Advanced Dulbecco Modified Eagle Medium (DMEM F12; Thermo Fisher Scientific, UK) enriched with Glutamax (1:100; Thermo Fisher Scientific, UK), 5% fetal bovine serum (Sigma-Aldrich, UK), penicillin-streptomycin (1:100; Life Technologies, UK), and Primocin (1:500; Sigma-Aldrich, UK). Once the filters turned completely transparent, indicating full saturation, the medium was aspirated and replaced with 1.65–2 mL of fresh medium. The plates were incubated overnight in a humidified chamber at 37°C with 95% air and 5% CO_2_ to ensure stabilization. Freshly sectioned lung slices (250 μm in PBS) were then placed on the Millicell filters and incubated under the same conditions for acclimatization. The medium was changed daily, with 1 mL removed and replaced each time until the experiment concluded.

### 2.8. Tissue processing and sectioning

Lung slices were carefully detached from the Millicell filters and transferred onto embedding cassette inserts, which were then sealed in jars filled with 4% paraformaldehyde and immersed overnight at 4°C. For *in vivo* studies, left lungs were excised and fixed in excess (five volumes) of 4% formaldehyde overnight at 4°C. After fixation, samples were transferred into 10 volumes of 70% ethanol and stored at 4°C for a maximum of 7 days. The samples were processed through a graded ethanol series (70%, 80%, 80%, 100%, 100%, and 100%), followed by three xylene washes and three paraffin washes before embedding in paraffin blocks using an HistoStar™ Embedding Workstation (ThermoFisher Scientific, UK). Paraffin blocks were sectioned to 4 μm thickness using a microtome (RM2125, Leica Biosystems, UK), and sections were placed on charged glass slides (J3800AMNZ, ThermoFisher Scientific, UK). Slides were dried overnight at 37°C. Following drying, sections were deparaffinized in citroclear and rehydrated through a graded ethanol series (100%, 100%, 80%, 80%, 70%, 50%, and 0%) for 3 min each. Antigen retrieval was conducted at 110°C for 2 min in citrate buffer (0.1 M citrate C9999, Sigma-Aldrich, UK, 0.05% TWEEN 20 P1379, Sigma-Aldrich, UK, pH 6.0) in a Decloaker Chamber (Biocare Medical, USA). After cooling for 20 min, the slides were removed from the citrate buffer.

### 2.9. Immunohistochemistry

Immunohistochemical (IHC) staining was carried out using the Real EnVision Detection System HRP/DAB for Rabbit/Mouse (Dako, UK), following the manufacturer’s instructions. After antigen retrieval, slides were rinsed in PBS for 3 min and blocked for 1 h at room temperature with Mouse-on-Mouse Blocking Reagent (MKB-2213, Vector Laboratories, USA) for mouse antibodies. The slides were then incubated overnight at 4°C with a diluted primary antibody in antibody diluent (ab64211, Abcam, UK). The primary antibodies used were mouse anti-phospho-histone H2AX (ser139) (γH2AX, 1:1000 dilution; 05-636, Millipore, UK) and rabbit anti-53BP1 (4937, 1:1000 dilution; Cell Signaling Technology, USA). The following day, slides were washed 3 times for 5 min each in PBS and incubated with the polymer reagent from the Dako kit for 1 h at room temperature. After a final wash in PBS (3 × 5 min), the stained sections were dehydrated in a graded ethanol series (50–100%) and treated with xylene before mounting with DPX. Samples were scanned using the Aperio ScanScope CS digital slide scanner (20× objective with 2× doubler; Leica Biosystems, UK) and analyzed with ImageScope Software (v11.2.0.780, Aperio, Leica Biosystems, UK).

ImageScope was used to manually count positively-stained cells under each experimental condition (80–250 cells/nuclei per field of view). Two independent experiments were conducted for each condition, with four fields of view analyzed per experiment.

### 2.10. Immunofluorescence (IF)

Deparaffinized tissue sections (4 μm) on slides were washed in PBS 3 times and blocked for 1 h at room temperature using a blocking buffer containing 5% goat serum (S26, Millipore, UK), 2% bovine serum albumin (HT110132, Sigma-Aldrich, UK), and 0.5% Triton X-100 (437002A, BDH Chemicals, UK) in PBS. Tissues were stained overnight at 4°C with primary antibodies (mouse anti-γH2AX and rabbit anti-53BP1, 1:100 dilution) in the blocking buffer. After three washes with PBS (3 × 5 min), slides were incubated in a dark chamber at room temperature for 1 h with either Alexa Fluor 488 goat anti-mouse immunoglobulin G (IgG) (A21422, Life Technologies, UK) or Alexa Fluor 555 goat anti-rabbit IgG (A11008, Life Technologies, UK), both diluted at 1:400 in blocking buffer. Following counterstaining and mounting with Fluoromount medium containing DAPI (0.1 μg/mL; ThermoFisher Scientific, UK), coverslip edges were sealed with nail polish. Slides were stored in the dark at 4°C for up to 7 days before microscopic evaluation.

IF images were captured using an LSM 710 point-scanning confocal microscope (Carl Zeiss, Germany) with a 63 ×/1.40 NA Oil DIC M27 Plan-ApoChromat objective. DAPI was excited with a 405 nm laser, and emission was captured within a range of 410–507 nm. Alexa Fluor 488 was excited at 488 nm, with emission captured between 495 nm and 573 nm, and Alexa Fluor 555 was excited at 561 nm, with emission captured between 568 nm and 697 nm. Confocal images were compiled into a maximum-intensity projection using Zen software (Zeiss, UK).

### 2.11. Statistical analysis

Data are expressed as mean ± SEM unless otherwise specified. Normality was assessed using the Kolmogorov–Smirnov test in GraphPad Prism (Dotmatics, UK). Where data demonstrated a normal distribution (as per the Kolmogorov–Smirnov test), statistical comparisons were performed using analysis of variance or *t*-tests in GraphPad Prism. All statistical analyses were exploratory, and no adjustments were applied for multiple comparisons. A *P* ≤ 0.05 was considered statistically significant, with the following significance thresholds: *P ≤ 0.05, **P ≤ 0.001, ***P ≤ 0.0001. Non-significant (*P* > 0.05) results were denoted as NS.

## 3. Results

### 3.1. Development of the organotypic PCLSs model

**Figure 1:**
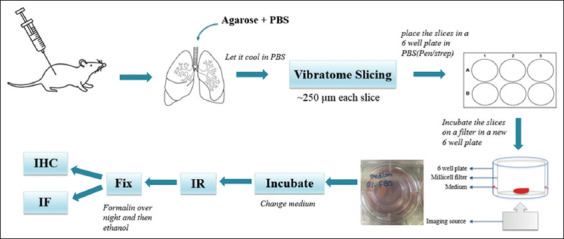
Overview of the mouse precision-cut lung slice model. Female C57/BL6 mice are euthanized, and their lungs are dissected and perfused with agarose, then left to solidify at 4°C. The left lung is separated and immersed in warm agarose before vibratome slicing. The lung is sectioned into 250 μm slices, which are placed in phosphate-buffered saline and individually transferred into wells of a pre-prepared 6-well plate containing a Millicell filter and tissue culture medium. The tissue slices are incubated overnight at 37°C in 95% air and 5% carbon dioxide to acclimate before treatment. Following treatment, the slices are fixed, stored in 70% ethanol at 4°C (up to 7 days), then processed, embedded in paraffin wax, sectioned, and stained by immunohistochemistry and immunofluorescence.

**Figure 2:**
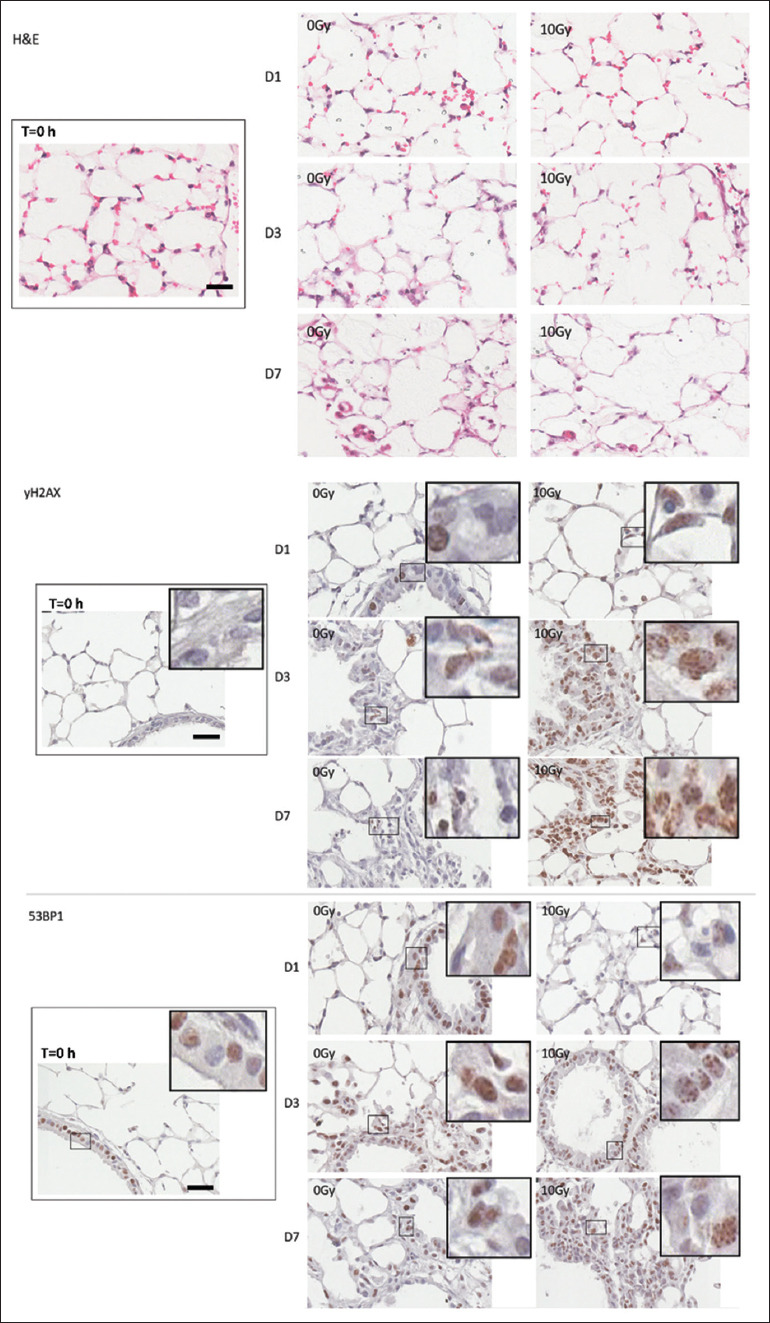
Lung structure and cellular integrity in tissue slice culture. Slices were incubated for up to 7 days. Lung slices were irradiated with 10 Gy on days 1, 3, and 7, and fixed 2 h after irradiation. Control slices were fixed immediately after vibratome sectioning (T = 0 h). Sections were stained by γH2AX and 53BP1 by immunohistochemistry or by hematoxylin and eosin. Scale bars = 15 μm; Insets show images enlarged by 2×.

We optimized our PCLS protocol based on methods initially developed for cancer xenograft tissues ( Figure 1 and Table 1).^10^,^11^ Throughout the 7-day culture period, alveolar structures remained mostly intact, although some disruption in bronchi epithelial cells was observed starting on day 3, with more extensive changes noted by day 7. These observations are consistent with findings from other studies.^12^ Morphological alterations were similar in both unirradiated and irradiated PCLS (10 Gy), as shown in Figure 2 (hematoxylin and eosin staining). To evaluate cell viability, we exploited the fact that ionizing radiation induces deoxyribonucleic acid (DNA) damage, which activates various acute adenosine triphosphate-dependent metabolic responses, including the redistribution of nuclear 53BP1 into distinct foci^13^-^15^ and the phosphorylation of histone variant H2AX at Ser-139 (γH2AX).^16^ Cells within the PCLCs maintained metabolic activity for up to 7 days, exhibiting a robust DNA damage response, as evidenced by the rapid and strong staining of both γH2AX and 53BP1 foci following irradiation (2 h post 10 Gy). In contrast, only sporadic staining was seen in mock-irradiated samples (Figure 2). Although PCLCs remained viable for at least 7 days, we restricted our PCLS experiments to a maximum of 2 days in culture to preserve the three-dimensional organotypic characteristics of the lung.

**Table 1:** Summary of sectioning and culture conditions for mouse precision-cut lung slices

Sectioning and culture conditions	Details
Preparation	
Time for agarose to solidify	15 min
Position of the lung	Posterior glued to slicing tray
Time limit for slicing (from dissection to incubation of slices)	≤45 min
Vibratome	
Angle of the blade	17°
Speed	0.60 mm/s
Amplitude	3.0 mm
Thickness of slices	250 µm
Culture conditions	
Filter	Millicell Biopore hydrophilic PTFE cell insert, 0.4 μm pore size 30 mm height
Medium	Advanced DMEM F12
Glutamax	1:100
Penicillin-streptomycin	1:100
Fetal bovine serum	5%
Primocin	1:500
Volume of medium	1.65–2 mL
Changing the medium	Remove and replace 1 mL of medium every day

### 3.2. Radiation-induced residual DNA double-strand breaks (DSB) in the PCLSs model

A single unrepairable DNA DSB can be lethal and lead to cell death.^17^ Consequently, cells rapidly activate mechanisms to resolve and repair these lesions.^18^,^19^ However, at cytotoxic radiation doses, a fraction of DSBs can persist 24 h after exposure, often referred to as residual, unrepaired, unrepairable, or long-lived DSBs.^20^-^26^ The presence of residual DNA DSBs 24 h post-irradiation serves as a key indicator of radiosensitivity and toxicity,^23^,^27^-^29^ and this can be quantified through IHC staining for γH2AX or 53BP1.^13^,^16^,^30^,^31^

**Figure 3:**
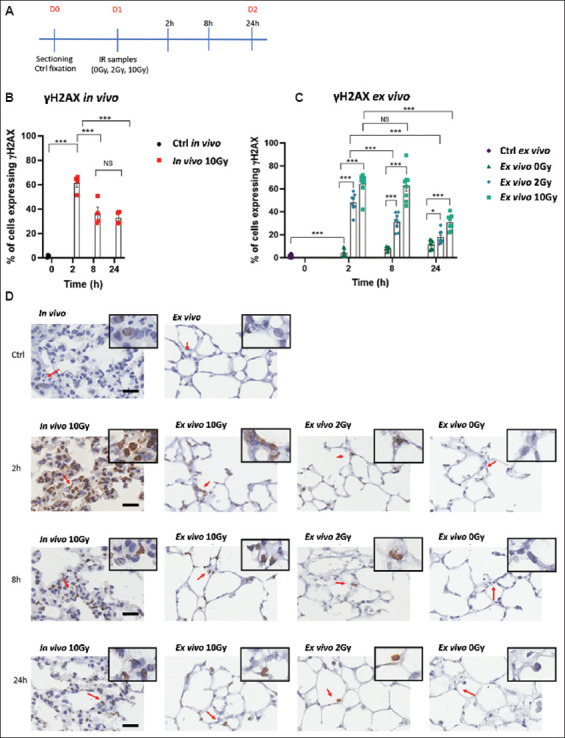
Phosphorylated histone H2AX (γH2AX) expression following *ex vivo* or *in vivo* irradiation. (A) Schematic of the precision-cut lung slice experimental protocol. (B) Mean number of γH2AX-positive cells in lung sections from mice irradiated *in vivo* (10 Gy) and fixed 2, 8, or 24 h later. (C) Mean number of γH2AX-positive cells in precision-cut lung slices irradiated *ex vivo* (0, 2, 10 Gy) and fixed 2, 8, or 24 h later. Control *ex vivo* samples (Ctrl) were fixed immediately after slicing (without culturing). (D) Representative immunohistochemical images of γH2AX-stained samples (Scale bars = 15 μm; Insets show images enlarged by 2×). Error bars represent the standard error of the mean. Notes: NS: Not significant; *p<0.05, **p<0.001, ***p<0.0001.

In our study, PCLS were exposed to either a low (2 Gy) or high (10 Gy) dose of ionizing radiation (Figure 3A), and residual DNA damage was assessed 24 h later via γH2AX staining (Figure 3C). Although individual γH2AX foci were detectable in some cells after irradiation, accurate counting was challenging due to the small size of the nuclei in alveolar cells, the predominant cell type in the lung (Figure 3D). As a result, the data are presented as the proportion of cells exhibiting γH2AX positivity^32^-^35^ (Figure 3C). Control PCLS exhibited only sporadic γH2AX staining (Figure 3C and D), but this increased after 24 h in culture (Figure 3C). Radiation exposure significantly elevated γH2AX staining at both low (2 Gy) and high (10 Gy) doses compared to controls (0 Gy, Figure 3C). In addition, γH2AX staining analyzed at earlier time points (2 and 8 h) indicated significant DSB repair in PCLS following irradiation (Figure 3C).

In a related experiment, mice were subjected to 10 Gy whole-thorax irradiation, which is the maximum non-lethal dose for this strain.^36^-^40^ This treatment also resulted in a notable proportion of γH2AX-positive cells (35%) 24 h post-irradiation (Figure 3B), a value comparable to that observed in *ex vivo* PCLS (35%) (Figure 3C). However, unlike *ex vivo* PCLS, the percentage of γH2AX-positive cells *in vivo* did not significantly decrease between 8 and 24 h post-treatment, possibly indicating a faster DSB repair process *in vivo* following irradiation (Figure 3B and C).

Similar to γH2AX, counting 53BP1 foci in alveolar cells was unreliable due to their small nuclei. However, the larger nuclei of bronchi epithelial cells allowed for more accurate counting of individual 53BP1 foci. This method provided clear evidence of DNA DSB repair in both *in vivo* and *ex vivo* models (Figure 3A-C), with the number of 53BP1 foci returning to near control levels 24 h after irradiation in both models (Figure 3A and B).

**Figure 4:**
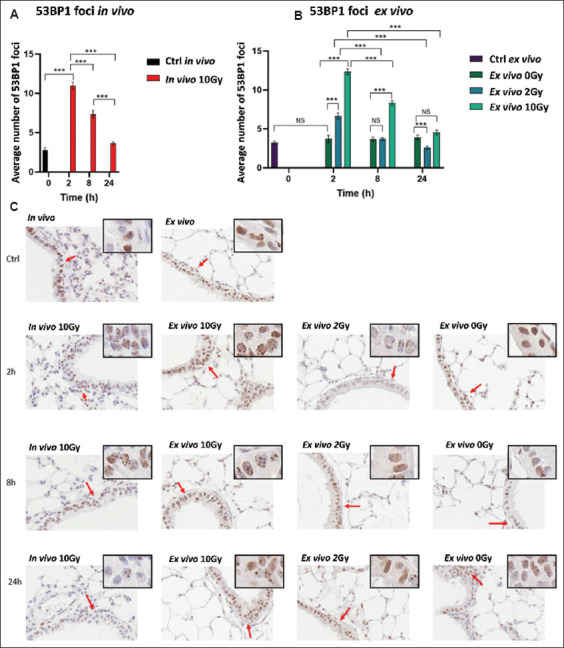
p53-binding protein 1 (53BP1) nuclear foci in bronchi epithelial cells following *ex vivo* or *in vivo* irradiation. The experimental outline is shown in Figure 3A. Mean 53BP1 nuclear foci counts in bronchi epithelial cells in lung sections either from mice irradiated *in vivo* (A) (10 Gy) or *ex vivo* (B) (0, 2, 10 Gy), with fixation 2, 8, or 24 h post-irradiation. Control *ex vivo* samples (Ctrl) were prepared from precision-cut lung slices fixed immediately after slicing, without culturing. (C) Representative images of 53BP1-stained samples. Error bars represent the standard error of the mean. Scale bars = 15 μm; Insets show images enlarged by 2×. Notes: NS: Not significant; *p<0.05, **p<0.001, ***p<0.0001.

Notably, differences were observed between the *in vivo* and *ex vivo* models concerning the extent of residual DNA damage, as indicated by γH2AX-positive staining in alveolar cells versus 53BP1 foci counts in bronchi epithelial cells (Figures 3 and 4). This observation prompted further investigation in the context of a DNA DSB repair inhibitor.

### 3.3. Effects of DNA-protein kinase (PK) inhibition on residual DNA DSBs

**Figure 5:**
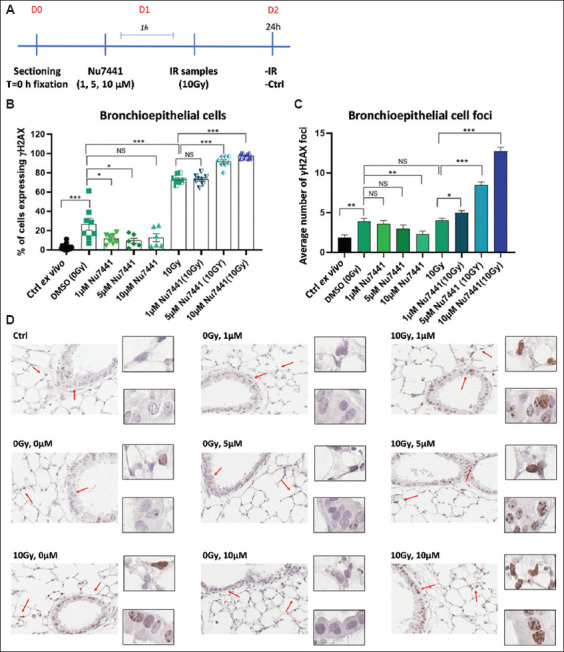
Phosphorylated histone H2AX (γH2AX) expression in bronchi epithelial cells of precision-cut lung slices (PCLSs) 24 h after treatment with NU7441 and ionizing radiation. (A) Experimental outline: PCLSs were incubated with dimethyl sulfoxide (DMSO) or NU7441 (1, 5, 10 μM) for 1 h before irradiation (0 or 10 Gy), and fixed 24 h post-irradiation. Control *ex vivo* samples (Ctrl, T = 0 h) were prepared from lung slices fixed immediately after slicing, without culturing. (B) Mean number of γH2AX-positive bronchi epithelial cells. (C) Mean number of γH2AX foci per nucleus. (D) Representative immunohistochemical images. Error bars represent the standard error of the mean. Scale bars = 15 μm; Insets show images enlarged by 2×. Notes: NS: Not significant; *p<0.05, **p<0.001, ***p<0.0001.

Non-homologous end joining is the primary pathway for DNA DSB repair^41^ and relies on DNA-protein kinase (PK) activity.^42^ We utilized NU7441, a selective DNA-PK inhibitor with limited clinical applications,^43^ in the PCLS model both independently and in combination with ionizing radiation (10 Gy) (Figure 5A).

As previously reported, irradiation alone resulted in increased γH2AX positivity in bronchi epithelial cells (Figures 5B and D), but did not significantly elevate residual DNA DSBs as measured by γH2AX foci (Figure 5C). The discrepancy between these two assessments may stem from additional factors that can raise γH2AX levels, including apoptosis, oxidative stress, hypoxia, and cell cycle stages.^44^ Unexpectedly, compared to untreated controls, NU7441 alone led to a reduction in both γH2AX positivity and the number of γH2AX foci, indicating that the background levels of γH2AX were partially dependent on DNA-PK activity.

**Figure 6:**
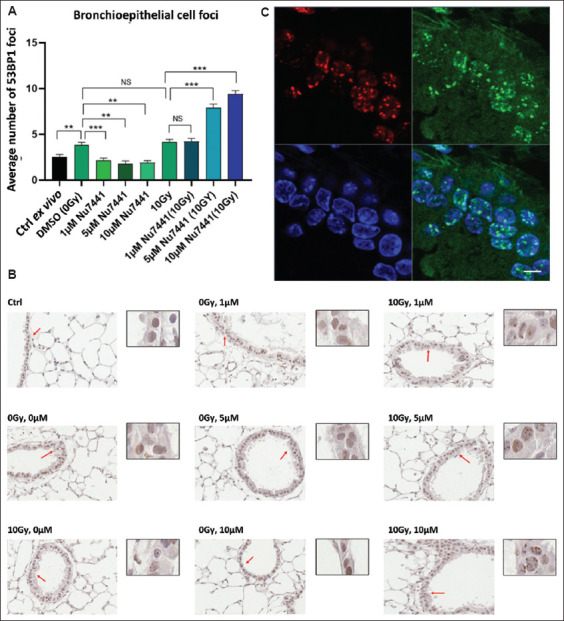
p53-binding protein 1 (53BP1) nuclear foci levels in bronchi epithelial cells of precision-cut lung slices 24 h after treatment with NU7441 and ionizing radiation. The experimental outline is shown in Figure 5A. (A) The mean number of 53BP1 foci per nucleus. (B) Representative 53BP1 immunohistochemical images, with red arrows indicating enlarged regions. (C) Dual immunofluorescent staining for phosphorylated histone H2AX (γH2AX) (green) and 53BP1 (red) foci. Top left: 53BP1, top right: γH2AX, bottom left: DAPI, bottom right: Overlay of γH2AX/53BP1/DAPI. Error bars represent SEM. Scale bars = 15 μm; Insets show images enlarged by 2×. Notes: *p<0.05, **p<0.001, ***p<0.0001.

The combination of NU7441 and irradiation resulted in a higher proportion of cells exhibiting γH2AX positivity (Figure 5B), particularly at increased NU7441 concentrations (Figure 5B). Conversely, NU7441 treatment led to a dose-dependent increase in radiation-induced residual DNA DSBs, as reflected in the number of γH2AX foci (Figure 5C). Similar trends were noted for 53BP1 foci staining (Figure 6A and B), suggesting that both markers are effective for detecting residual DNA DSBs.^23^

Both γH2AX and 53BP1 foci are recognized as indirect markers of DNA DSBs,^15^ and our findings corroborate this, with comparable counts of γH2AX and 53BP1 nuclear foci in PCLS sections (Figures 5 and 6). To further examine this, we performed dual-label IF on irradiated PCLS slices, which showed colocalization of γH2AX foci and 53BP1 foci (Figure 6C). However, a detailed coexpression study (Pearson correlation) was not performed.

### 3.4. Residual DNA damage in irradiated PCLSs derived from severe combined immunodeficient mice

**Figure 7:**
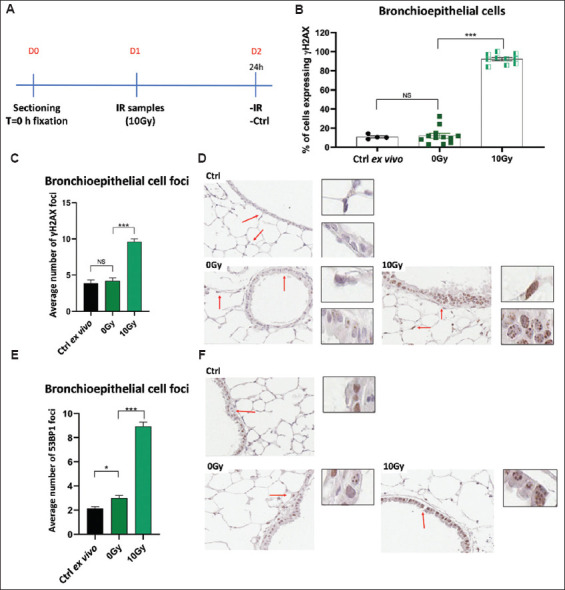
p53-binding protein 1 (53BP1) and phosphorylated histone H2AX (γH2AX) expression in precision-cut lung slices from deoxyribonucleic acid-protein kinase-deficient severe combined immunodeficient mice 24 h after *ex vivo* irradiation. (A). Experimental outline. (B) The mean number of γH2AX-positive cells per section. (C) Mean number of γH2AX foci per nucleus, with representative images shown in panel (D). (E) Mean number of 53BP1 foci per nucleus, with representative images shown in panel (F). Scale bars = 15 μm; Insets show images enlarged by 2×. Notes: *p<0.05, **p<0.001, ***p<0.0001.

Severe combined immunodeficient mice, which lack DNA-PK enzymatic activity,^12^ provide a model for indirectly comparing the genetic loss of DNA-PK activity with pharmacological inhibition using NU7441 (Figure 7).

Twenty-four hours after irradiating PCLS from SCID mice (10 Gy) (Figure 7A), we observed a significant increase in both the percentage of cells expressing γH2AX (Fig. 6B) and the number of γH2AX foci (Figures 7C and D) in irradiated samples compared to unirradiated (0 Gy) controls. These results align with the established role of DNA-PK in the repair of DNA DSBs.^42^ Likewise, the count of 53BP1 foci (Figures 7E and F) was significantly elevated in irradiated SCID PCLS, indicating a higher level of residual DNA DSBs.

The results from PCLS studies involving SCID lungs (Figure 7) are consistent with those obtained from NU7441 treatment, particularly at higher concentrations (≥5 μM) (Figures 5 and 6). These findings demonstrate that both genetic and pharmacological inhibition of DNA-PK results in an increase in residual DNA DSBs, further reinforcing DNA-PK’s critical role in DNA DSB repair and its potential as a radiosensitizer.^42^,^43^,^45^

## 4. Discussion

The objective of this study is to establish *ex vivo* PCLS as an organotypic model for investigating the effects of radiation and drug-radiation combinations. Although the PCLS remained viable for at least 7 days in culture, we observed significant histological changes by day 2, likely due to the trauma associated with tissue slicing. Methodologically, identifying distinct γH2AX or 53BP1 foci in the small nuclei of alveolar cells proved challenging when using standard IHC with a DAB chromogen. In contrast, the larger nuclei of bronchioalveolar cells allowed for clearer visualization of nuclear foci, suggesting that assessing residual DNA DSBs in these cells may provide more sensitive measurements compared to alveolar cells. Notably, the levels of radiation-induced residual DNA DSBs, as indicated by γH2AX or 53BP1 foci, were largely consistent between our *ex vivo* and *in vivo* investigations. These findings are in line with a recent *in vivo* study involving a different DNA-PK inhibitor, which also demonstrated a significant increase in radiation-induced residual DNA DSBs in lung tissue.^46^

Organotypic models offer several advantages.^46^ They maintain the three-dimensional architecture of tissues, which better simulates *in vivo* conditions and the physiological functions of organs.^47^ This preserved structure ensures that different cell types retain their spatial arrangement, facilitating cellular communication within a tissue context. In addition, organotypic models preserve important cell-matrix interactions, which influence cell behavior and fate.^48^ Furthermore, these models present an ethical alternative to animal testing,^49^ reducing ethical concerns while providing a relevant platform for drug testing within a biological framework.

However, organotypic models also present several challenges.^46^ The setup process is intricate and requires multiple steps, including tissue harvesting, processing, and meticulous monitoring of culture conditions, making it labor-intensive. Importantly, these models typically lack functional vasculature and the normal perfusion found in living tissues, which can limit their physiological relevance in certain contexts. At present, no universally accepted protocol for establishing, maintaining, or assessing organotypic cultures, which hinders their scalability for larger studies, such as drug screening.^50^ While some organotypic models demonstrate stability over extended periods, others may exhibit limited viability or degradation of cellular and extracellular matrix components over time.^50^

In summary, organotypic culture models are valuable tools in biomedical research, providing a more accurate representation of tissue biology compared to traditional two-dimensional cultures. Nonetheless, researchers must address the complexities and challenges associated with these models. By recognizing both their benefits and limitations, scientists can make informed decisions about the appropriate application of organotypic cultures in their research, ultimately advancing our understanding of human biology and improving therapeutic strategies.

## 5. Conclusion

Our studies, employing both pharmacological and genetic inhibition of DNA-PK activity, demonstrate increased residual DNA damage following radiation in the PCLS model. These findings suggest that the organotypic PCLS model could have broader applications, such as in target validation and early drug discovery, particularly in cases where high-quality, *in vivo* active inhibitors have not yet been developed.

## Data Availability

Data used in this work are available from the corresponding author upon reasonable request.
